# Eu-Doped BaTiO_3_ Powder and Film from Sol-Gel Process with Polyvinylpyrrolidone Additive

**DOI:** 10.3390/ijms10094088

**Published:** 2009-09-17

**Authors:** Margarita García-Hernández, Antonieta García-Murillo, Felipe de J. Carrillo-Romo, David Jaramillo-Vigueras, Geneviève Chadeyron, Elder De la Rosa, Damien Boyer

**Affiliations:** 1 Instituto Politécnico Nacional, CICATA Unidad Altamira, Km. 14.5, Carretera Tampico-Puerto Industrial Altamira, C.P. 89600 Altamira, Tamps, Mexico; E-Mails:margarciah@ipn.mx (M.G.-H.);angarciam@ipn.mx (A.G.-M.); 2 Instituto Politécnico Nacional, CIITEC, Cerrada CECATI S/N Col. Sta. Catarina, Del. Azcapotzalco, México D.F. 02250, Mexico; E-Mail:djaramillo@ipn.mx (D.J.-V.); 3 Université Blaise Pascal, Laboratoire des Matériaux Inorganiques, CNRS-UMR 6002, 63177 Aubière, France; E-Mails:genevieve.chadeyron@univ-bpclermont.fr (G.C.);dboyer@chimtp.univ-bpclermont.fr (D.B.); 4 Centro de Investigaciones en Óptica, A.P. 1-948, 37150 León, Gto., Mexico; E-Mail:elder@cio.mx (E.D.l.R.)

**Keywords:** BaTiO_3_, europium, sol-gel, films, luminescence

## Abstract

Transparent BaTiO_3_:Eu^3+^ films were prepared via a sol-gel method and dip-coating technique, using barium acetate, titanium butoxide, and polyvinylpyrrolidone (PVP) as modifier viscosity. BaTiO_3_:Eu^3+^ films ~500 nm thick, crystallized after thermal treatment at 700 ºC. The powders revealed spherical and rod shape morphology. The optical quality of films showed a predominant band at 615 nm under 250 nm excitation. A preliminary luminescent test provided the properties of the Eu^3+^ doped BaTiO_3_.

## 1. Introduction

In recent years, lanthanide-doped ultrafine and nanocrystalline oxide materials have been widely investigated due to their optical properties, which make them promising candidates for applications in optoelectronic devices and flat panel displays [1–3]. Additionally, perovskite-structure materials are attractive as host matrices for rare earth doping because they present promising properties in integrated light-emission devices, field emission displays (FEDs), all-solid compact laser devices operating in the blue-green region, and positive coefficient (PCT) resistors [4–7]. Research has been particularly active for binary oxides systems such as cubic Y_2_O_3_ [8] and Lu_2_O_3_ [9], mainly doped with the technologically important Eu^3+^ ions. These investigations have been extended to mixed oxides and, in particular, the optical spectroscopy of BaTiO_3_ powders doped with Eu^3+^ has been extensively studied [10–14]. The formation of Eu^3+^ doped BaTiO_3_ films is attractive due to its revealed luminescent properties [15]. BaTiO_3_ thin films have previously been prepared by different methods such as MOCVD [16], sputtering [17], electrophoretic deposition [18] and sol-gel [19–21]. Among these methods, the sol-gel route has been intensively studied because, in general, this process is flexible enough to produce ceramic powders, fibers, and monoliths, as well as advantageously elaborate films of complex oxides [22–25]. This method implies the formation of a colloidal (‘sol’) solution which is traditionally based on dissolved organometallic molecular precursors. In the hydrolysis and polycondensation reactions, the macromolecular oxides species M-O-M occurs via oxolation [26]. The formation of M-O-M products degrades the stability of the precursors and fails to yield the final ceramic products: precipitate, gel or stable colloidal solution. The hydrolysis problem has been investigated by the addition of glacial acetic acid and acetylacetone to Ti(OR)_4_ [27,28].

The present study describes a modified sol-gel process for preparing europium doped BaTiO_3_ films using monosubstituting agents like acetic acid [29] and acetylacetone [11] to change the precursors formed in the conventional sol-gel process [30], and using polyvinylpyrrolidone (PVP), which has proved to have significant advantages in the elaboration of uniform non-doped BaTiO_3_ thick films [31–33]. Juan Li *et al.* reported the elaboration of BaTiO_3_:Eu^3+^ crack-free films by sol-gel process [15]; nevertheless, there have been no reports related on BaTiO_3_:Eu^3+^ sol-gel films in presence of PVP. The aim of this study is to provide the ability to disperse europium ions in the BaTiO_3_ film structure and investigate the effect of PVP on structure, morphology, thickness and luminescence properties.

## 2. Results and Discussion

### 2.1. Chemical Studies

**Figure 1.**
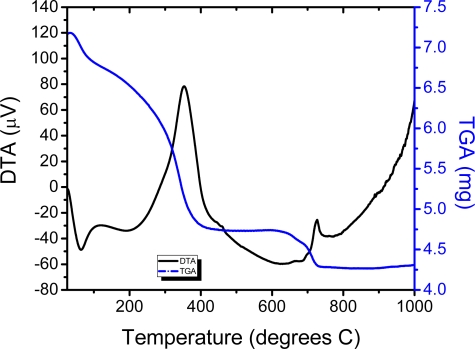
DTA and TGA curves for BaTiO_3_:Eu xerogel powder.

TG and DTA curves of the europium-doped BaTiO_3_ powders dried at 100 °C for 24 h are presented in Figure 1. In the first temperature region up to 200 °C, the endothermic peak situated at 115 °C results from the evaporation of alcohol and excess water. One endothermic peak situated at ~350 °C represents the decomposition of organic compounds. The third weight loss at 700–750 °C, associated with an exothermic peak, points out the transformation of amorphous decomposition products in BaTiO_3_. During decomposition, there is not significant loss of titanium according to Madarász [34] for the TiO(acac)_1.0_ hydroxo complexes for samples with acetylacetone content higher than 0.49. The acetylacetone anion deficiency is compensated with hydroxide ions considered products of hydrolysis (acac-Ti, + H_2_O-Ti.. + acacH) according to the following reaction [35]:

$$\text{M}\;{\left(\text{OR}\right)}_{4}+(\text{AcAc})\text{H}\to \text{M}{\left(\text{OR}\right)}_{3}(\text{AcAc})+\text{R}-\text{OH}$$

where M represent the metal atom M and ROH the leaving group. According to the XRD results, crystallization of barium titanate starts at 700 °C Moreover, the weight loss is minimal and the weight remans unchanged afterwards. It was stated that Ba^2+^ ions in the A site are mainly replaced by rare earth elements [36]. Eu^3+^ ions (0.098 nm) are most probably replaced Ba^2+^ (0.156 nm) cations rather than Ti^4+^ (0.065 nm). When Eu^3+^ was introduced to BaTiO_3_, three Ba^2+^ sites were substituted by two Eu^3+^ neighbors to maintain electrical neutrality, and so one vacancy was created, then the composition expected is Ba_(1-0.05)_Eu_0.05_TiO_3_, as observed by Rath [14].

**Figure 2.**
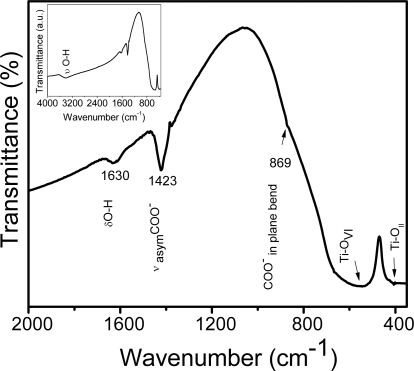
FTIR spectrum of BaTiO_3_:Eu^3+^ sol-gel powder calcined at 700 °C.

Figure 2 shows the FTIR spectrum of BaTiO_3_:Eu powders, calcined at 700 °C for 2 h. The IR spectrum consisted mainly of three regions: the first region (Figure 2 inset) shows bands at 3,428 and 1,630 cm^−1^, which are due to the OH stretching vibration (υ) and OH deformation vibration (δ), respectively, arising from the water and isopropanol present in the porous structure of the barium titanante xerogel. The second region corresponds to the absorption bands at 1,423 and 869 cm^−1^, characteristic for the symmetrical vibrations and bending vibrations (in plane) of COO- groups arising from two types of ligands (the acetylacetone and acetic acid).The third region, 600-380 cm^−1^, represents the characteristic infrared absorptions of the Ti-O vibrations. The band situated around 565 cm^−1^ is due to TiO_6_ stretching vibration connected to the barium [37]. Finally, the peak at 414 cm^−1^ can be attributed to normal TiO_II_ bending vibrations [38].

**Figure 3.**
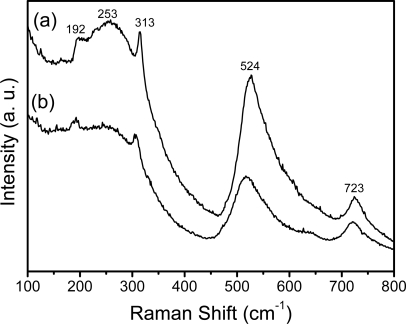
Raman spectra of BaTiO_3_:Eu^3+^ powder (a) and film (b).

In order to complete the investigations of the local structure of the sol-gel BaTiO_3_:Eu^3+^ derived powders (Figure 3a) and films (Figure 3b), Raman spectra were used to measure the samples annealed at 700 °C. Both recorded spectra contain characteristic bands: (a) one weak band [A1(TO), E(LO)] at 192 cm^−1^, and (b) two intense broad bands A1(TO1) at ~253 cm^−1^ and A1(TO4) at 524 cm^−1^, with sharp peaks at ~313 cm^−1^ (TO3-LO3) and for the LO4 band at ~723 cm^−1^. Referring to Amami *et al.* [39], the sharp peaks situated at ~185 and ~235 cm^−1^ are associated with the cubic phase. Nevertheless, it is accepted by many researchers that the Raman peak at around 260 cm^−1^, which is somewhat variable in relation to particles size, shape, and aggregation, is due to the characteristics of tetragonal BaTiO_3_ [40–43]. Finally, the origin of the above described bands in the cubic phase has been disputed due to the presence of Raman modes in this phase, indicating that it does not have perfect cubic symmetry but has small distortions [44]. By using the Raman-active modes discussed above, it was observed that the tetragonal phase is present in both BaTiO_3_ powder and film. However, the Raman band positions in the powder spectrum do not exactly match the peak positions in the films, which could be attributed to the internal stress from the surface tension in the nanocrystals [45].

### 2.2. HT-XRD and XRD Studies

**Figure 4.**
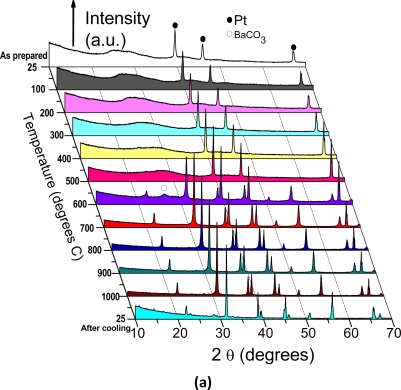

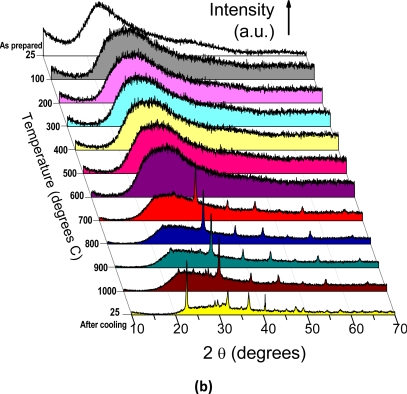
HT-XRD patterns of BaTiO_3_:Eu^3+^ powders (a) and films (b). Diffraction peaks related to the platinum ribbon are noted with a cross.

HT-XRD is used to follow *in situ* the formation of the BaTiO_3_:Eu^3+^ oxide from the BaTiO_3_ precursor gel as powder and film (see Figures 4a and b, respectively). The first scan is the pattern for the as-synthesized xerogel at room temperature, along with the intense peaks at 2θ values of about 40, 46 and 67 (results from the Pt sample holder). In the scan corresponding to 600 °C (Figure 4a), it is shown that some barium carbonate is formed during the decomposition of the precursor into the BaTiO_3_.

**Figure 5.**
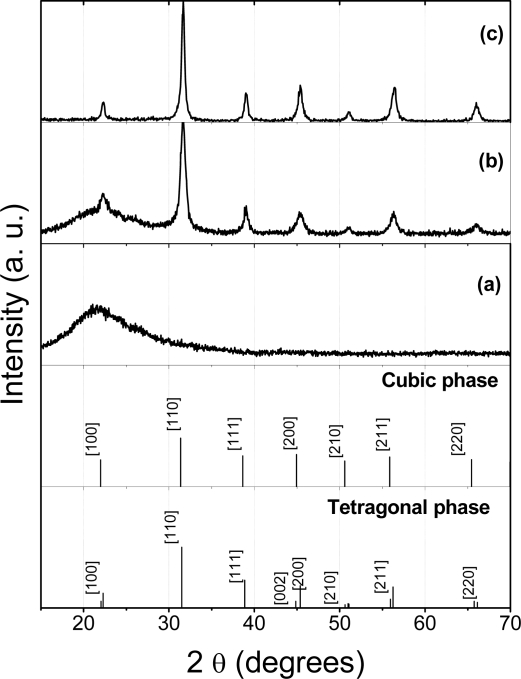
XRD patterns of BaTiO_3_:Eu^3+^ film calcined at 500 °C (a), 700 °C (b) and powder calcined at 700 °C (c).

This carbonate decomposes between 650 and 700 °C. This led to our conclusion that pure BaTiO_3_ is fully crystallized after 2 h at 700 °C. Figure 5c shows the full XRD 2θ range of the crystallized powders, showing that two distinct peaks characterize BaTiO_3_ tetragonal structure [46]. Additionally, *in situ* HT-XRD experiments were performed to understand the phase stability of nanocrystalline BaTiO_3_:Eu^3+^ sol-gel films. Figure 4b shows the multiple plots of the barium titanate gel films scanned in air at various temperatures; from room temperature to 1000 °C, and again at room temperature after cooling. The gel film patterns indicate presence of an amorphous structure character up to 600 °C (Figure 5a) and the crystallization of BaTiO_3_:Eu^3+^ after the films were heated to 700 °C (Figure 4b and Figure 5b), in agreement with DTA analyses. The patterns of films calcined from 700 °C to 1000 °C are characterized by nanocrystalline BaTiO_3_ samples. Due to the very broad diffraction peaks attributed to the presence of the support or to the film thickness, it is not a sensitive enough technique to easily distinguish between ferroelectric tetragonal and paraelectric pseudocubic structure (Figure 5b). Nevertheless, it was found that after cooling from 1000 °C to room temperature, the BaTiO_3_:Eu^3+^ films transform from (1 1 0) orientation dominated to (1 0 0) dominated orientation, probably due to the textured densified films. The rates of cooling are mainly responsible for the high preferential orientation achieved in the BaTiO_3_ films. The transformation of orientation has been also observed in the case of PB_x_T layers films deposited on MgO (1 0 0) substrates [47].

### 2.3. Microscopy Observations

**Figure 6.**
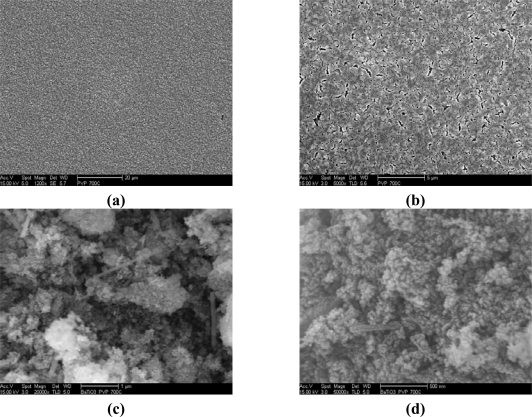
SEM micrographs of BaTiO_3_:Eu^3+^ of films (a), (b) and powders (c), (d) calcined at 700 °C.

**Figure 7.**
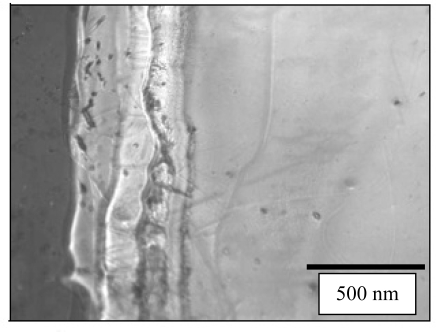
Cross section SEM micrograph of BaTiO_3_:Eu^3+^ film calcined at 700 °C.

The morphology of BaTiO_3_:Eu^3+^ powder and film calcined at 700 °C was investigated by SEM and is shown in Figure 6. The SEM images of BaTiO_3_:Eu^3+^ powders shown in Figures 6a and 6b reveal closely-packed fine equiaxed particles, about 100 nm in size. Aditionally, non-equiaxed powders (nanorod shaped structures) reaching ~800 nm in length were observed. On the other hand, equiaxed particles consisted of the approximately spherical type. For example, the shape of BaTiO_3_:Eu^3+^ powders at 700 °C results in a combination of non-equiaxed and equiaxed morphology due to the coexistence of the tetragonal and cubic phase. From the surface images of BaTiO_3_:Eu^3+^ films shown in Figures 6c and 6d, the microstructures were homogeneous, continuous and crack-free. At high magnification, as shown in Figure 6d, there was no evidence of cracks; however, the presence of pores can be associated with the presence of PVP. Kozuka *et al.* [48] have reported that when the film is heated directly at 700 °C, the decomposition of PVP and the crystallization of the film may occur concurrently. This can lead to crystallization with much less densification, which provides higher porosity and smaller tensile stress. Profilometry is a very common method of post-process measurement of films thickness [49]. The thickness of BaTiO_3_:Eu^3+^ calcined at 700 °C thin film was about 500 nm is in agreement with the determined by cross section SEM, as can be observed in Figure 7.

### 2.4. Luminescence Properties

**Figure 8.**
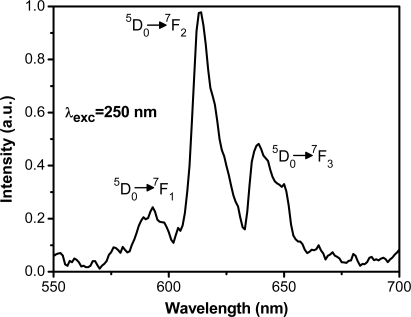
PL emission spectrum of BaTiO_3_:Eu^3+^ film annealed at 700 °C under UV excitation. The inset shows a picture of the strong emission.

The room temperature photoluminescence emission spectrum of BaTiO_3_:Eu^3+^ (5 mol %) film in the range of 550–700 nm and excited at 250 nm wavelength is shown in Figure 8. The red emission from the film is easily seen to the naked eye when excited with 254 nm from UV lamp (see the inset of Figure 8). Peaks centered at 595, 615 and 645 nm are assigned to ^5^D_0_ →^7^F_1,_ ^5^D_0_ →^7^F_2_ and ^5^D_0_ →^7^F_3_, respectively, arising from the lowest excited ^5^D_0_ level into the split by the crystal field ^7^F_J_ (where J = 0, 1, 2, 3, 4, 5, 6) as observed by other authors and is in agreement with results reported for bulk Eu^3+^ doped cubic yttria [50–51].

In most cases, transitions to the higher laying levels (^7^F_5_, ^7^F_6_) are difficult to detect due to their low intensity [52]. The ^5^D_0_ → ^7^F_1_ band originates from magnetic-dipole transition and, in this case, the change of the crystal field strength has very little influence on it. The dominant peak is observed around 615 nm (^5^D_0_ → ^7^F_2_) and is attributed to the forced electric-dipole transition allowed only at low symmetries with no inversion center. Its intensity is sensitive to the local structure surrounding the Eu^3+^ ions. Thus the ratio R = (^5^D_0_ →^7^F_2_)/(^5^D_0_ →^7^F_1_) > 1 suggest that Eu^3+^ occupy sites with low symmetry. The origin of these transitions (electric dipole or magnetic dipole) from emitting to terminating levels depends upon the site where Eu^3+^ is located in the host lattice, and the type of these transitions is determined by the selection rules. It must be mentioned that the signal emitted of BaTiO_3_:Eu^3+^ films are not very strong, probably due to the final thickness, influencing directly the suitable effective value of providing high emission [11].

## 3. Experimental Section

### 3.1. Experimental Procedure

Europium doped BaTiO_3_ films were prepared using the sol-gel process and the dip-coating technique. The starting materials were: barium acetate, Ba(CH_3_COO)_2_ [Aldrich], titanium butoxide, Ti(C_4_H_10_O)_4_ [Aldrich], europium III chloride, EuCl_3_ [Alfa Aesar] acetylacetone, C_5_H_8_O_2_ [Aldrich], acetic acid, C_2_H_4_O_2_, [99.8%, Fermont], distilled water, H_2_O, isopropyl alcohol, C_3_H_7_OH [99.9%, Fermont] and polyvinylpyrrolidone, PVP; FW: 630,000 g mol^−1^ [Alfa Aesar]. Barium acetate and europium chloride were dissolved in water in the following molar ratio: Ba:Eu:H_2_O [1:0.05:40]. Titanium butoxide was mixed with acetylacetone in a molar ratio [1:1], and the reaction was continued under reflux at 60 °C for 6 h. PVP was dissolved in isopropyl alcohol in a molar ratio [1:18] for 2 hours at room temperature. The molar ratios were calculated with respect to Ba. The titanium and barium solutions were mixed and stirred for 2 h. Thereafter, the PVP solution was added to the previous solution drop by drop under vigorous magnetic stirring for 2 h at room temperature. In this step, a transparent and stable BaTiO_3_:Eu^3+^ sol was formed. Uniform coatings of BaTiO_3_:Eu^3+^ were dip coated on high polished and carefully cleaned silica substrates (Herasil from Heraeus®) with a constant withdrawal speed of 5 cm min^−1^ for five cycles. BaTiO_3_:Eu^3+^ films were dried at 100 °C between each coating under O_2_ flow for 10 min. In this step, one film was taken for HT-XRD studies. The BaTiO_3_:Eu^3+^ films were finally calcined at 700 °C for 10 min. The remaining solution was dried in order to obtain a xerogel at 100 °C for 24 h (xerogel was analyzed in HT-XRD). After drying, the xerogel was thermally treated at 700 °C for 2 h in order to densify and crystallize the sol-gel powders.

### 3.2. Apparatus

The xerogel was investigated by Thermogravimetric (TGA)-Differential Thermal (DTA) and High-Temperature X-Ray Diffraction (HT-XRD) analyses. The thermograms were recordered from 23 °C to 1000 °C using a Mettler Toledo TGA/SDTA 851e apparatus at a scan rate of 2 °C min ^−1^ in a flux of nitrogen. The HT-XRD studies were carried out on a Philips Xpert Pro diffractometer operating with the Cu Kα radiation and equipped with a high temperature chamber, over a temperature range from 25 to 1000 °C in air atmosphere. The structural phases of the crystallized powder and film were recorded on a Siemens D5000 powder diffractometer using the Bragg–Brentano configuration and the Cu Kα radiation. The IR transmittance spectra were recorded on the crystallized powders calcined at 700 °C using a FTIR 2000 Perkin-Elmer in the range 4,000-400 cm^−1^, with the aim of completing DRX and DTA-TGA experiments. In order to determine the powder microstructure and quality of the derived BaTiO_3_:Eu^3+^ crystallized sol-gel films, SEM images were obtained using a JEOL 3200 scanning electron microscope (SEM) with a field emission gun operating at 15 and 8 kV. The thickness of BaTiO_3_ films was determined via an Alpha-step IQ profilometer (Tencor Instruments). The fluorescence emission spectra for BaTiO_3_:Eu^3+^ film as obtained with a modular Spectra Pro (Acton Research) fluorometer with a PM tube R955 (Hamamatsu).

## 4. Conclusions

The sol-gel method and dip-coating techniques have been successfully employed to prepare BaTiO_3_:Eu^3+^ (5 mol%) powder and film incorporating a viscosity modifier (PVP) in the sol. Both systems exhibited crystalline BaTiO_3_ phase at 700 °C within 2 h as revealed the HT-XRD studies. The powders were mainly spherical with some rod shapes; however, the films presented good surface morphology as detected by SEM. The obtained films exhibit the room temperature photoluminescence of the europium ions, with the predominant band at 615 nm (^5^D_0_→^7^F_2_ transition). The obtained thickness (~500 nm) of BaTiO_3_:Eu^3+^ films must be optimized to be promising for luminescent applications.
